# 

**Published:** 1818-11

**Authors:** 


### BOOKS PREPARING FOR PUBLICATION.

On the 4th of December will be published The Medical Intelligencer, or Monthly Index to the Periodical Publications of the present day; also to the Transactions of Medical Societies, and all works containing medical, chemical, and chirurgical communications, or reviews of medical works; a list of new publications, and other miscellaneous information relating to medical subjects.

Another edition of Dr. Armstrong's book on Scarlatina, Measles, &c. is printing.

New editions of Dr. Armstrong's works on Typhous Fever, and on Puerperal Fever, will also shortly appear.

### CATALOGUE OF MEDICAL BOOKS.

Memoirs of the Life and Doctrines of the late John Hunter, Esq. By Joseph Adams, M.D. The second edition, corrected. 8vo. 7s. 6d.

Medical Sketches; by George Kerr. 12mo. 4s.

A Manual of Practical Anatomy, for the use of Students engaged in Dissection. 12mo. 9s.

Elements of Medical Chemistry; by M. P. Orfila. Translated from the French.

Physiological and Medical Researches into the Causes, Symptoms, and Treatment, of Gravel. Translated from the French of F. Majendie, M.D.

The Medical Cabinet, or Physician's Pupil's Directory; containing— 1. Pharmacopœia Londinensis. 2. Translation of ditto into English, with Notes and Indexes. 3. Pharmacopœia Guyensis. 4. Dr. Clarke's Conspectus of the London, Edinburgh, and Dublin Pharmacopœias. 12mo. 16s. bound.

